# Characterization of the soybean KRP gene family reveals a key role for GmKRP2a in root development

**DOI:** 10.3389/fpls.2023.1096467

**Published:** 2023-01-27

**Authors:** Binhui Guo, Lin Chen, Lu Dong, Chunhong Yang, Jianhua Zhang, Xiaoyan Geng, Lijuan Zhou, Li Song

**Affiliations:** ^1^ Joint International Research Laboratory of Agriculture and Agri-Product Safety, Institute of Agricultural Science and Technology Development, Co-Innovation Center for Modern Production Technology of Grain Crops, Yangzhou University, Yangzhou, China; ^2^ Basic Experimental Teaching Center of Life Science, Yangzhou University, Yangzhou, China; ^3^ College of Forestry, Co-Innovation Center for the Sustainable Forestry in Southern China, Nanjing Forestry University, Nanjing, China

**Keywords:** soybean, root development, drought, KRP, cell cycle

## Abstract

Kip-related proteins (KRPs), as inhibitory proteins of cyclin-dependent kinases, are involved in the growth and development of plants by regulating the activity of the CYC-CDK complex to control cell cycle progression. The KRP gene family has been identified in several plants, and several KRP proteins from *Arabidopsis thaliana* have been functionally characterized. However, there is little research on KRP genes in soybean, which is an economically important crop. In this study, we identified nine *GmKRP* genes in the *Glycine max* genome using HMM modeling and BLASTP searches. Protein subcellular localization and conserved motif analysis showed soybean KRP proteins located in the nucleus, and the C-terminal protein sequence was highly conserved. By investigating the expression patterns in various tissues, we found that all *GmKRPs* exhibited transcript abundance, while several showed tissue-specific expression patterns. By analyzing the promoter region, we found that light, low temperature, an anaerobic environment, and hormones-related *cis*-elements were abundant. In addition, we performed a co-expression analysis of the *GmKRP* gene family, followed by Gene Ontology (GO) and Kyoto Encyclopedia of Genes and Genomes (KEGG) set enrichment analysis. The co-expressing genes were mainly involved in RNA synthesis and modification and energy metabolism. Furthermore, the *GmKRP2a* gene, a member of the soybean KRP family, was cloned for further functional analysis. GmKRP2a is located in the nucleus and participates in root development by regulating cell cycle progression. RNA-seq results indicated that GmKRP2a is involved in cell cycle regulation through ribosome regulation, cell expansion, hormone response, stress response, and plant pathogen response pathways. To our knowledge, this is the first study to identify and characterize the KRP gene family in soybean.

## Introduction

In the life cycle of plants, progression through the cell cycle, including cell division and cell expansion, is fundamental to plant growth. The cell cycle is strictly regulated by cyclin-dependent kinases (CDKs) with specific cyclin partners. Cyclin-dependent kinase inhibitor/interactors (CKIs) bind to CDKs, cyclins, or the CDK–cyclin complex and inhibit their association or activity to provide another cell cycle regulation level (Mendenhall, 1998; Sherr and Roberts, 1999; Godínez-Palma et al., 2017). Plant CKIs have two distinct families: KIP-RELATED PROTEINS (KRPs) and SIAMESE-RELATED proteins (SMRs). Plant KRP and SMR members have been identified and analyzed in several species, including *Arabidopsis*, tobacco, rice, maize, and tomato (De Veylder et al., 2001; Jasinski et al., 2002; Bisbis et al., 2006; Guo et al., 2007; Xiao et al., 2017).

The root system plays crucial roles during plant growth and development processes, including nutrient and water absorption, anchoring and mechanical support, and nutrient storage function. Root development is a very complex process that undergoes precise regulation (Motte et al., 2019). *KRP* plays an important role in root development. In *KRP2*-overexpressing *Arabidopsis* plants, the cell size is enlarged and the number of lateral roots is reduced by ~50% (De Veylder et al., 2001; Verkest et al., 2005a), and the knockout mutant *krp2* shows increased lateral root density (Sanz et al., 2011). *KRP4* is involved in root meristem maintenance in *Arabidopsis thaliana* (Singh et al., 2016). *KRP5* plays a positive role in the cell elongation of *Arabidopsis* primary roots by promoting the progression of endoreduplication (Jégu et al., 2013; Wen et al., 2013). In addition, the roots are the main interface between biotic and abiotic stress factors. Recently, progress has been made on how KRPs or SMRs are involved in biotic and abiotic stress at the molecular level. Moderate drought quickly induces SMR1 (SIAMESE-RELATED1) expression levels, which then inhibits the growth of roots and leaves by inhibiting cell division and affecting the vitality of the meristem in *Arabidopsis* (Dubois et al., 2018). ZmSMR4 is not only induced by PEG at the transcription level but also plays a role in seed development (Li et al., 2019). KRP members are involved in RKN (root knot nematode) resistance by inhibiting giant cells (Vieira and de Almeida Engler, 2017; Coelho et al., 2017). The expression of multiple cell cycle regulators in maize root tips is regulated by a variety of environmental factors, such as salt stress and osmotic stress (Zhao et al., 2014).

Furthermore, the regulation of KRP activity can occur at both the transcriptional and posttranslational levels. For example, *Arabidopsis* endoreduplication tissues or mitotically dividing cells may prefer different KRP members (Ormenese et al., 2004; Menges et al., 2005). KRP2 strong overexpression of *Arabidopsis* transgenic lines inhibits the endoreduplication cycle, whereas weak lines promote the endoreduplication cycle (De Veylder et al., 2001; Verkest et al., 2005a). A 26S proteasome inhibitor increases the stability of the KRP2 protein *in vitro*. Additionally, KRP2 degradation needs to be phosphorylated by the CDKB1;1 complex (Verkest et al., 2005a; Jakoby et al., 2006). Phosphorylation of the KRP from *Medicago truncatula* (Mt) protein by the recombinant calmodulin-like domain protein kinase (MsCPK3, from cultured alfalfa cells) results in enhanced inhibition of CDK function (Pettko-Szandtner et al., 2006). NtKIS1b, from *Nicotiana tobacco*, a spliced variant of NtKIS1a, antagonizes the roles of NtKIS1a, which act in CDK activity inhibition (Jasinski et al., 2002). In summary, the modulation of KRP activity at the posttranslational modification is achieved by phosphorylation, proteolysis, histone modification, and splicing (Churchman et al., 2006; Li et al., 2016; Liu et al., 2016).

Soybean is an economically important crop that is the main source of vegetable protein, edible oil, and feed. The demand for soybean production continues to grow with a rapidly growing population (Foley et al., 2011). *KRP* gene family information has been characterized in several plant species, but little research has been conducted in soybean. Investigating the roles of *KRP* genes enables us to understand regulation of soybean root growth and development mediated by cell cycles regulators then help to increase soybean yield. In this study, KRP sequences were identified and used for phylogenetic analysis, resulting in the classification of KRP proteins into three groups. Conserved sequences and putative functional motifs, including novel soybean-specific motifs, were identified. Furthermore, family-wide gene expression patterns were evaluated using the qPCR method. Finally, functional studies of *GmKRP2a* demonstrated that it is a positive root elongation factor in soybean and *Arabidopsis*. These results provide experimental proof for GmKRP2a, which could be a target for the modification of soybean root growth.

## Materials and methods

### Identification of *KRP* genes in soybean genome and gene structure analysis

Pfam ID (PF02234) searches and BLAST using *A*. *thaliana* KRP2 protein sequences (NP_190632) as queries were performed in the *Glycine max* reference genome (a4.v1 version) to identify members of the soybean *KRP* gene family; all hits were classified as candidate *GmKRPs*. The exon/intron organizations of the genomic sequences, CDS, and protein sequences for all KRPs were retrieved from Phytozome (V13, https://phytozome.jgi.doe.gov). The gene structure schematic diagram of the KRPs was drawn and visualized with TBtools (Chen et al., 2020).

### Sequence alignment, phylogenetic tree analysis, and calculation of Ka/Ks values

Sequence alignments of multiple KRPs from *Glycine max* and other 6 dicots (*Phaseolus vulgaris*, *Vigna unguiculata*, *Arabidopsis thaliana*, *Lotus japonicus*, *Medicago truncatula*, and *Cicer arietinum*) and 3 monocots (*Oryza sativa*, *Sorghum bicolor*, and *Zea mays*) were performed using MUSCLE. The unrooted phylogenetic tree was made by the neighbor-joining method using MEGA 11 (Tamura et al., 2021). The reliability of an inferred tree was confirmed with bootstrap analysis performed with 1000 replications. The non-synonymous (Ka) and synonymous substitution (Ks) rate ratios of the paralog pairs were calculated using the TBtools program (Chen et al., 2020). The divergence time (T) was calculated following T = Ks/(2 x 6.1 x 10^−9^) x 10^−6^ Mya (Lynch and Conery, 2000).

### Protein profile, conserved domain, and SNP analysis

The protein lengths, molecular weights, and isoelectric points of the GmKRPs were analyzed using the ProtParam online tool (Gasteiger et al., 2005). Protein subcellular localization was predicted using the WoLF PSORT online tool (Horton et al., 2007). All KRP protein sequences were analyzed to identify conserved protein motifs (Motif scan) using the ‘Multiple EM for Motif Elicitation’ (MEME) program (Bailey et al., 2009). The parameters were as follows: zero or one occurrence per sequence, motif width ranges of 6–50 amino acids, and 10 as the maximum number of finding motifs. Each of the sequences had an E-value of less than 10. The PEST domains were searched (http://emboss.bioinformatics.nl/cgi-bin/emboss/epestfind) (threshold score >+5.0) (Rogers et al., 1986). All SNP datasets located in the coding regions were extracted from whole genome re-sequencing datasets using SNPViz (https://soykb.org/SNPViz2/) as described by Langewisch et al. (2014). Non-synonymous SNPs (coding missense variant) were further analyzed and listed.

### *GmKRP2a* gene cloning and vector construction

The full-length coding region of *GmKRP2* was amplified from the root tissue of soybean ‘Williams82’ using gene-specific primers (GmKRP2-F: 5′-caccATGGAGATGGCTCAGGTTAAG-3′ and GmKRP2-R: 5′-TGGCTTCAATTTAACCCACTCG-3′) and Phusion high-fidelity DNA polymerase (Cat#: F530S, Thermo Scientific, Waltham, MA, USA). PCR products with the size of 556 bp were cloned into the Gateway pENTR-D-TOPO vector (Cat#: K240020, Invitrogen, Carlsbad, CA, USA). After sequence verification, it was recombined into the destination vector pMDC83 (Curtis and Grossniklaus, 2003) *via* LR reactions (Cat#: 11791020, Invitrogen, Carlsbad, CA, USA), which contained the 35S promoter of Cauliflower mosaic virus and fused with GFP at the N-terminal.

### Regeneration and molecular analysis of KRP2a-overexpressing plants

pMDC83-GmKRP2a::GFP was introduced into the *Agrobacterium rhizogenes* strain K599. Hairy roots infected with K599 containing the modified empty vector pMDC83 without the chloramphenicol resistance gene served as the control. Soybean transformation was performed using a previously reported protocol (Govindarajulu et al., 2008). Williams82 was used for hairy root transformation. After positive root selection using hygromycin (25 mg/L) in the medium, the transgenic roots were transferred to the new medium to determine the relative root growth length. At least 30 independent transgenic roots were used as biological replicates.

The pMDC83-GmKRP2a::GFP vector was introduced into *Agrobacterium tumefaciens* strain GV3101 and then transformed into wild-type Columbia-0 (Col-0) using the floral dip method (Clough and Bent, 1998). Putative transgenic lines were selected with 30 mg L^−1^ hygromycin on a half-strength MS medium that contained 1% sucrose and 0.8% agar. Three single-copy transgenic lines from the second generation were selected according to the segregation ratio. In this study, seedlings of Col-0 and transgenic plants were grown on half-strength MS medium under a long-day photoperiod (16 h light, 8 h dark), and the temperature was 22°C with 50–70% relative humidity. The hypocotyl and root lengths were measured at 5 and 10 days, respectively.

### Subcellular localization analysis

For the visualization of subcellular protein in *Arabidopsis* roots, 5-day-old transgenic seedlings harboring GFP fusion protein were visualized. The root tip was observed using a Leica SP8 spectral confocal microscope. For the cell size analysis, *Arabidopsis* roots were stained with 1 µg/ml propidium iodide. For the visualization of subcellular protein in *N*. *benthamiana* leaves, the coding sequence of GmKRP2a was fused to the C-terminal under the control of the CaMV 35S promoter in the transient expression vector pHB-mCherry, referred to as GmKRP2a-mCherry. The transient expression construct was introduced into *Agrobacterium* strain GV3101 and then used to infiltrate *N*. *benthamiana* leaves, as described previously (Lim et al., 2014). For GmKRP2a-GFP transgenic seedlings nuclear staining, the seedling was transferred to 4′,6-diamidino-2-phenylindole (DAPI, 5 mg/L) stain solution and incubated for 1 to 2 min at room temperature. For GmKRP2a-mCherry in tobacco leaves, the leaves were transferred to DAPI (5 mg/L) stain solution and incubated for 20 to 30 min at room temperature. After incubation, seedlings or leaves were placed on slides and then imaged with fluorescence microscope Confocal imaging analysis was performed using a Zeiss LSM 880 NLO laser scanning confocal microscope.

### Flow cytometric analysis of soybean hairy roots and leaves of *Arabidopsis*

Young *Arabidopsis* rosette leaves were chopped in LB01 nuclear dissociation buffer. The mixtures were filtered with a 40-μm cell filter, then the released nuclei were centrifuged (1000 rcf, 1 min) and resuspended in LB01 buffer. DAPI (final concentration of 4 μg/ml) was used to stain the nuclei before analysis with a flow cytometer (FACS LSRFortessa, BD, USA). For hairy roots, 2–3 cm of each root tip was cut in LB01 buffer to prepare the nuclear dissociation solution for flow cytometric detection and analysis of the content of nuclear DNA. The results were analyzed using Modfit 5.0 software. At least 1x10^5^ nuclei were used for the analysis.

### RNA-seq and reference-based transcriptome assembly

Total RNA was isolated from 100 mg hairy roots with the plant RNA Purification reagent (Cat#:74904, Invitrogen, UAS) following the manufacturer’s protocols. The RNA quantity was checked with NanoDrop2000 (Thermo Scientific, USA). RNA integrity was analyzed using an Agilent 2100 Bioanalyzer (RNA Nano Chip, Agilent, Santa Clara, CA, USA). High-quality RNA (RIN ≥ 9, OD260/280 ≥ 1.8–2.2, OD260/230 ≥ 1.0, 28S:18S ≥ 1.0, > 1 μg)) was used for library construction. The TruSeq RNA sample prep kit (Cat#: RS-122-2101, Illumina, USA) was used to prepare the RNA-seq library, and the library was quantified with QuantiFluor^®^ dsDNA System. The paired-end RNA-seq sequencing library was sequenced with the Illumina NovaSeq6000 sequencer (2×150 bp read length, Illumina, Inc. San Diego, CA, USA).

The data were analyzed on the online platform of the Majorbio Cloud Platform (www.majorbio.com). Briefly, Fastp (https://github.com/OpenGene/fastp) was used to obtain high-quality clean reads with default parameters. Clean reads were then separately aligned to the reference genome (*Glycine max Wm82*.*a4*.*v1*), with orientation mode using HISAT2 (http://ccb.jhu.edu/software/hisat2/index.shtml) software (Kim et al., 2015). The mapped reads of each sample were assembled using StringTie (https://ccb.jhu.edu/software/stringtie/) in a reference-based approach (Pertea et al., 2015). KEGG pathway analysis was carried out using Goatools (https://github.com/tanghaibao/Goatools) and KOBAS (http://kobas.cbi.pku.edu.cn/home.do) (Xie et al., 2011).

### Plant materials treatments and gene expression analysis with quantitative RT-PCR

Soybean Williams 82 seeds were germinated on a Petri dish lined with moist filter paper and then seedlings were transferred to half-strength MS solution grown in a growth chamber under a 10-h photoperiod at 25°C/22°C (day/night) temperature and 50 % relative humidity. The plants at the vegetative 1 stage were transferred to half MS solution containing 15% PEG6000 or 150 mM NaCl for 24 h and 48 h, respectively. The roots and first trifoliolate leaves from 5 plants were harvested and used for the *GmKRP* gene expression analysis. Samples were frozen in liquid nitrogen immediately after harvest and kept at -80°C for total RNA isolation. Soybean seeds were collected from the field.

Total RNA was isolated from soybean hairy roots or *Arabidopsis* seedlings or soybean seeds using an RNApure Plant Kit (CW0588S, CWBIO, Beijing, China), and cDNA was synthesized using HiScript III qRT SuperMix for qPCR (Cat#: R323-01, Vazyme, China). Gene-specific primers for quantitative PCR and the size of PCR products are listed in **Supplementary Table 1**. Real-time PCR amplification was carried out with the CFX connect Real-Time PCR detection system (Bio-Rad) using a ChamQ Universal SYBR qPCR Master Mix (Q711-02). *Actin11* (Glyma18g290800) was used as the housekeeping gene to normalize the expression level. All qPCR analyses had three biological replicates and two technical replicates. Differences in variables were analyzed by *t*-test. The differences were considered to be significant at p < 0.05 or p < 0.01.

## Results

### Identification and phylogenetic analysis of the soybean KRP family

To identify soybean KRP members, BLAST and PFAM (PF02234: cyclin-dependent kinase inhibitor) searches were performed using *Arabidopsis KRP* genes as guides. Nine putative GmKRP loci were found by searching the *Glycine max* reference genome (Wm82.a4.v1). The gene name, gene ID, protein information, and putative subcellular location are listed in **Table 1**. Among the nine putative GmKRP proteins, the amino acid sequences of GmKRPs were relatively consistent in size (ranging from 168 to 224 amino acids), but the PI (isoelectric point) showed wide variation ranging from 5.44 to 9.37. The subcellular location of this gene family was analyzed to better understand its biological function. All GmKRP proteins were predicted to localize in the nucleus (Cell-PLoc 2.0 and CELLO), providing GmKRPs a possibility to play roles in the cell cycle. In addition, all GmKRPs were hydrophilic proteins, and their instability coefficient was over 40, indicating that the KRP family of proteins is unstable.

**Table 1:** Physicochemical properties and subcellular localization of predicted soybean KRP proteins.

Gene ID	Gene Name	Length (aa)	pI	MW (kDa)	Instability index	Aliphatic index	Hydrophilicity/Hydrophobicity	Subcellular localization
Glyma.01G185400.1	GmKRP1a	198	5.44	22.08	54.25	70.00	Hydrophilicity	Nuclear
Glyma.11G056700.1	GmKRP1b	205	5.63	22.85	58.40	67.07	Hydrophilicity	Nuclear
Glyma.05G085000.1	GmKRP2a	184	5.75	20.62	47.73	70.00	Hydrophilicity	Nuclear
Glyma.17G175700.1	GmKRP2b	168	5.64	19.05	52.71	56.96	Hydrophilicity	Nuclear
Glyma.08G354300.1	GmKRP3	201	7.82	22.82	52.15	65.12	Hydrophilicity	Nuclear
Glyma.20G198800.1	GmKRP4	224	9.37	25.54	54.22	55.40	Hydrophilicity	Nuclear
Glyma.18G170800.1	GmKRP5	205	8.53	23.29	59.05	61.02	Hydrophilicity	Nuclear
Glyma.02G133700.1	GmKRP6	176	6.90	19.82	55.97	55.97	Hydrophilicity	Nuclear
Glyma.07G211300.2	GmKRP7	273	9.08	30.87	56.25	72.12	Hydrophilicity	Nuclear

**Figure 1:**
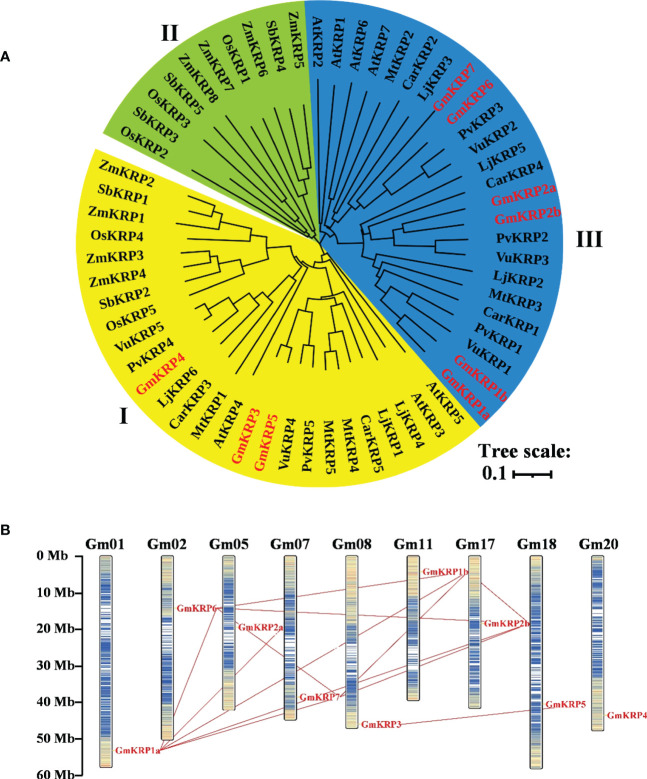
Molecular phylogenetic relationships of the KRP members in 10 different species based on the full-length sequences and chromosomal distribution and duplication analysis of the *GmKRP* genes. **(A)** The phylogenetic tree was constructed by the neighbor joining method with the MEGA11, and the bootstrap values are in percent (1000 replicates). Abbreviations: At, *Arabidopsis thaliana*; Gm, *Glycine max*; Pv, *Phaseolus vulgaris*; Vu, *Vigna unguiculata*; Lj, *Lotus japonicus*; Mt, *Medicago truncatula*; Car, *Cicer arietinum*; Os, *Oryza sativa*; Zm, *Zea mays* and Sb, *Sorghum bicolor*. **(B)** Paralogous of the *GmKRP* genes were mapped onto soybean chromosomes and connected with red lines.

To better assess the diversity of the *KRP* gene family beyond soybean, an unrooted phylogenetic tree was constructed using the Neighbor-joining method based on multiple KRP protein sequence alignments from dicot and monocot plants (**Figure 1A**). Nine soybean KRP genes were named according to the cluster of the KRP family, and the others were named based on their chromosomal location or previous reports (**Supplementary Table 2**). Accordingly, the multiple KRP sequences were divided into three major classes based on the phylogenetic tree. Class I contained 26 sequences from both monocotyledonous and dicotyledonous plants, including three soybean KRPs (GmKRP3, GmKRP4 and GmKRP5); Class II contained 16 sequences, only from monocotyledonous plants; Class III contained the remaining 24 sequences, specifically from dicotyledonous plants, including 6 soybean KRP members (GmKRP1a, GmKRP1b, GmKRP2a, GmKRP2b, GmKRP6 and GmKRP7). In addition, the *KRP* genes in the seven dicotyledons had closer relationships than those of the monocots, which was consistent with the evolutionary relationship between dicots and monocots. Furthermore, the KRPs from legumes (*Glycine max*, *Phaseolus vulgaris*, *Vigna unguiculata*, *Lotus japonicus*, *Medicago truncatula*, and *Cicer arietinum*) had closer relationships than those of genes from Cruciferae (*Arabidopsis thaliana*). These results prompt a hypothesis of the divergent evolution of KRP members in dicots and monocots.

### Gene structure, chromosome localization, and duplication analysis of the soybean KRP family

The exons and introns of the GmKRP genes were further analyzed by aligning the genomic sequence with the corresponding protein coding sequence. As illustrated in **Supplementary Figure 1**, three *GmKRP* genes had three exons (*GmKRP3*, *GmKRP 4*, *GmKRP 5*), while the rest contained four exons. The KRP members in other species contained three or four exons, except *OsKRP2*, *OsKRP6*, and *ZmKRP8*, which contained seven or two exons (**Supplementary Figure 1**). Moreover, the number and position of exons were conserved in classes I and III, although the UTR region varied. The last exon in all genes was the most conserved in terms of size, sequence, and encoding protein. In general, the gene structure showed sequence relatedness consistent with the phylogenetic tree based on the protein sequences.

Nine soybean *KRP* family members were distributed on different chromosomes (**Figure 1B**). We detected 12 fragment duplicated gene couples among *GmKRP* genes in the soybean genome. No tandem duplications were found in the GmKRP gene family. Therefore, segmental duplication was the main reason for the expansion of the *GmKRP* genes. The Ka/Ks ratio was further calculated to evaluate the selection pressure during evolution. Determining whether positive Darwinian selection was involved in *GmKRP* divergence following duplication and estimating the date of the duplication pairs, as shown in **Supplementary Table 3**, the ratio between different gene pairs varied from 0.178 to 0.413, indicating that the *GmKRP* genes primarily evolved under the influence of purifying selection. The duplication time among classes I and II varied from 140–257 Mya (million years ago). The duplication time of gene pairs in class I or class II was 68–83 Mya. However, the separation times of *GmKRP1a*/*GmKRP1b*, *GmKRP6*/*GmKRP7*, and *GmKRP3*/*GmKRP5* pairs varied from 10 to 14 Mya; this period was consistent with the latest twice whole genome duplication of soybean. These results uncovered that *GmKRP* expansion was derived from whole genome duplication, resulting in conserved domains and motifs.

### Conserved domains and motifs in soybean ICK/KRP protein

The alignment of the soybean KRP protein sequence showed that all GmKRP proteins had a highly conserved CID domain (**Supplementary Figure 2A**). However, the sequence composition at the N terminal showed wide variation. Moreover, 10 conserved sequence motifs were further identified using the MEME program, with an E-value less than 0.05 in the GmKRP amino acid sequence (**Supplementary Figure 1**). Motifs 1 and 2, located at the extremity of the C-terminal part of the proteins, were shared by all examining KRPs, including GmKRPs. The distribution of other motifs follows phylogenetic classification. The GmKRPs in class I contained motifs 3, 4, 5, 6, 8, and 9. The GmKRPs in class II contained motif 9 or 10. The GmKRPs in class III contained motifs 5, 6, 7, and 10. The sequence information for each motif is provided in **Supplementary Figure 2B**. In addition to the conserved motifs, PEST domains, which were reported to serves as a proteolytic signal, were found in GmKRP1a, 1b, 2b, and 6 (Rogers et al., 1986, **Supplementary Figure 2B**).

### *Cis*-element and co-expression network analysis of the GmKRP gene family

**Figure 2:**
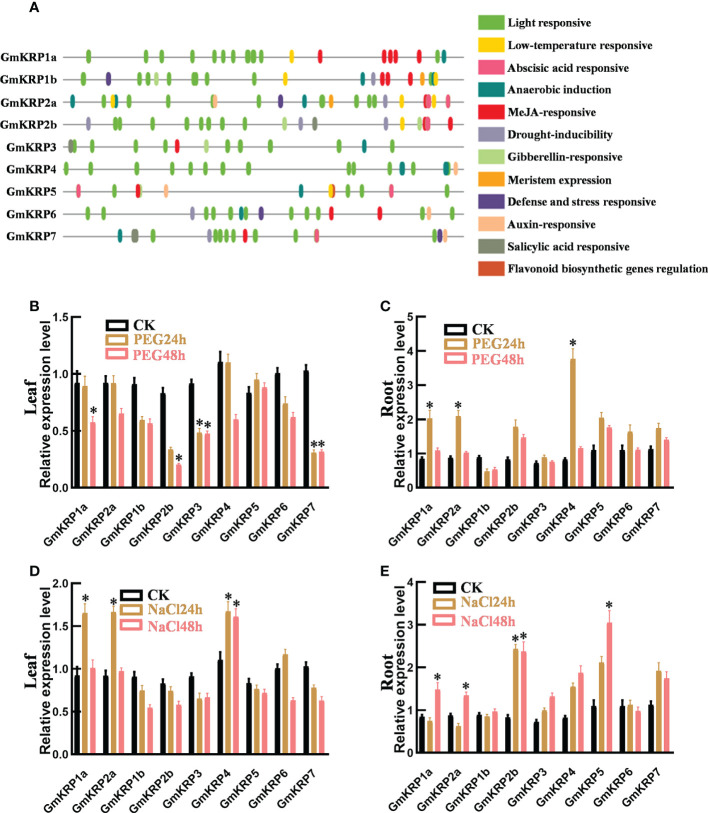
*Cis*-element analysis and expression patterns of *GmKRP* genes under different conditions. **(A)** *Cis*-element analysis on the promoter region of the *GmKRP* genes. The potential *cis*-regulatory elements in the 2 kb promoter regions were predicted by PlantCARE software. Different colors indicated the elements related to different functional categories. **(B-E)** Expression patterns analysis of the *GmKRP* genes in leaf and root under PEG stress **(B, C)**, in leaf and root under NaCl stress **(D, E)**. Data were collected from three independent biological samples per treatment and three technical replicates per samples. Error bars represent standard error. Asterisks indicates significant differences at 5% level respectively between treatments and control.

To further investigate the putative functions of the GmKRP genes, we analyzed the 2 kb promoter region of all *GmKRP* genes using PlantCARE web tools. As expected, Core *cis*-elements, TATA- and CAAT-box, were both found in all promoters in the promoter region. In addition, 12 types of *cis*-acting elements were found in the promoter region of the GmKRP genes (**Figure 2A**). These regulatory elements were mainly involved in abiotic stress responses (low temperature, anaerobic and drought) and hormone responses (methyl jasmonate, auxin, gibberellin, abscisic acid and salicylic acid). Among all the *cis*-elements, the light-responsive elements correspond to the highest frequency, and the soybean KRP gene is likely to participate in the stress response and light-induced development process in soybean growth. To investigate whether the expression of *GmKRP* genes was affected by abiotic stress, the expression levels of *GmKRP* genes were evaluated by qPCR analysis under abiotic stress (PEG and salt stress). Under PEG stress, the expression levels of *GmKRP1a*, *GmKRP2b*, *GmKRP3*, *and GmKRP7* were downregulated in leaves, while three genes involving *GmKRP1a*, *GmKRP2a*, and *GmKRP4* expression levels showed upregulated in root (**Figures 2B, C**). Under salt stress, several *GmKRP* genes were upregulated both in leaves and roots (**Figures 2D, E**).

A total of 862 co-expressed genes of the GmKRP family were retrieved from the soybean genome database. These co-expressed genes were used in underlying pathways that GmKRP may be involved in through Gene Ontology (GO) enrichment analysis. These co-expressed genes were mainly enriched in protein modification and phosphorylation, as well as Ras-genes (GO:0007265) and ARF, Adenosine diphosphate-ribosylation factor (GO: 0032011) signal transduction in the biological process (**Supplementary Figure 3A**). For molecular function terms, nucleotide binding, kinase activity, and kinase inhibitor activity were enriched in co-expressing genes. These results are consistent with the signal pathways involved in the regulation of the cell cycle by KRP and its molecular function prediction (**Supplementary Figure 3B**).

### Gene expression pattern of the GmKRP family in soybean tissues

**Figure 3:**
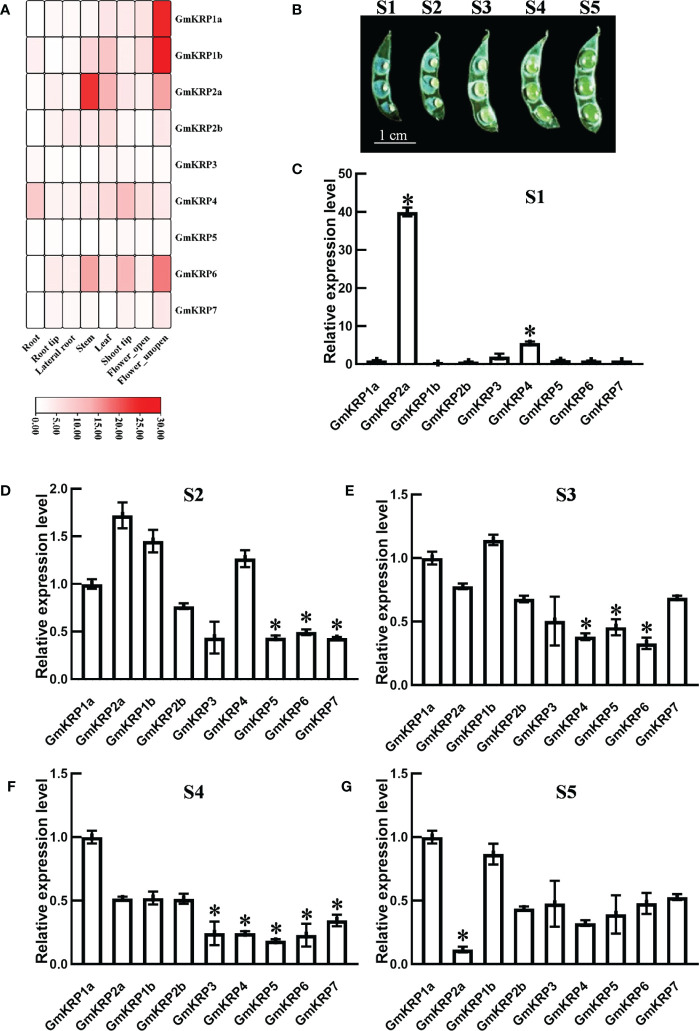
Expression patterns analysis of the *GmKRP* genes in various developmental stages and tissues of soybean. **(A)** Relative tissue expression levels of *GmKRPs* based on RNA-Seq data were used to construct the expression patterns by using the RPKM (Reads Per Kilobase Million) values. The color scale bars on the right display the expression levels of each gene. Red represents high expression levels, whereas white represents low expression levels. **(B-G)** Relative expression profile of the *GmKRP* genes during seed development by qRT-PCR. Bar=1 cm. The wide length of seed in S1: 0.1~0.14 cm, S2: 0.15~0.18 cm, S3: 0.18~0.25 cm, S4: 0.25~0.28 cm, S5:0.28~0.33 cm. The expression data are the average values ± SD of two independent reactions from three set of total RNA. The reference genes (*GmActin11*) used to normalize the *GmKRP* gene expression in each stage. Asterisks indicates significant differences at 5% level respectively between GmKRP1a and other GmKRP genes.

To gain insight into the tissue expression pattern of the GmKRP members, a soybean RNA-seq database was extracted, including eight tissues (root, root tip, lateral root, stem, leaf, shoot tip, open and unopen flower) (Libault et al., 2010). As illustrated in **Figure 3A**, *GmKRP2b*, *GmKRP3*, *GmKRP4*, *GmKRP5*, and *GmKRP7* showed relatively uniform expression in all tissues. *GmKRP4* showed relatively high expression in all tissues. Obviously, *GmKRP1a*, *GmKRP1b*, *GmKRP2a*, and *GmKRP6* were identified as having tissue-specific expression. *GmKRP1a* and *GmKRP1b* was highly expressed in the unopened flower. *GmKRP2a* was highly expressed in stems. *GmKRP6* showed predominantly expressed in the stem, shoot and unopened flower. The expression patterns of *GmKRPs* in five seed-development stages were further investigated using qPCR (**Figure 3B**). We found that GmKRP2a and GmKRP4 were highly expressed in early seed development stage (**Figure 3C**). In addition, the expression level of GmKRP2a was lower than that of other *GmKRP* genes at the late stage of seed development (**Figure 3D-G**). Overall, the tissue organ-specific expression pattern indicates that *GmKRP* transcripts are abundantly expressed in unopened flower and relatively weakly expressed in root.

### Genome-wide natural polymorphisms of the GmKRP gene family

The SNPs located in the *GmKRP* coding regions that may lead to function variation were retrieved from 775 soybean germplasms containing both wild and cultivated species using SNPViz software. A total of 52 non-synonymous substitution SNPs were observed in nine *KRP* genes. Non-synonymous substitution SNPs were absent in the protein-encoding region of *GmKRP1b* in any of the 775 soybean lines. Compared with the reference genome (Wm82.a4.v1), one and two non-synonymous substitution SNPs were found in *GmKRP2a* and *GmKRP3* in one or two wild soybean germplasms, respectively. Three to six non-synonymous substitution SNPs were found in the coding region of *GmKRP4*, *GmKRP5*, and *GmKRP7* in less than 20 germplasm resources. However, in *GmKRP1a*, *GmKRP2b*, and *GmKRP6*, more non-synonymous substitution SNPs (8–19 SNPs) were found, which come from more than 100 germplasm resources (**Supplementary Table 4**). These results indicate that *GmKRP1b* and *GmKRP2a* may play a highly conserved function during evolution because few non-synonymous substitution SNPs were found in soybean landraces and elite lines.

### GmKRP2a involved in root and hypocotyl elongation in transgenic *Arabidopsis* seedlings and soybean hairy roots

**Figure 4:**
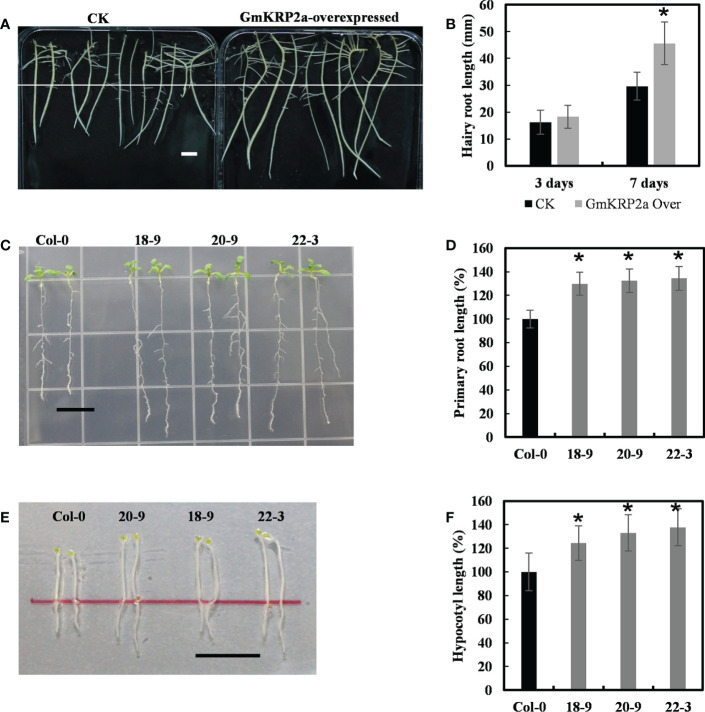
Overexpression of the GmKRP2a promotes root and hypocotyl elongation in soybean hairy root and *Arabidopsis*. **(A)** Comparison length of seven-day-old dark-grown soybean hairy root. Left: empty vector control; Right: GmKRP2a-overexpressing. **(B)** Statistical analysis of hairy root length of control and GmKRP2a-overexpressing root at the third and seventh days, respectively. **(C)** Ten-day-old wild type (Col-0) and GmKRP2a-overexpressing seedlings under 16 h light/8 h dark. **(D)** Statistical analysis of relative root length of wild type and GmKRP2a-overexpressing seedlings. **(E)** Five-day-old wild type (Col-0) and GmKRP2a-overexpressing seedlings under continues dark. **(F)** Statistical analysis of relative hypocotyl elongation of wild type and GmKRP2a-overexpressing seedlings. The Arabidopsis seeds were planted on the 1/2MS medium contain 2% sucrose and 1% Agar. 18D, 20D and 22D were 3 independent transgenic lines. n≥30, P<0.01, Bar=1cm. Data were shown from three independent experiments, and bars indicate SDs. *, P,0.01.

To further study the function of the *GmKRP* genes, one of the highly conservative gene, *GmKRP2a* gene was cloned from *Glycine max* Wm82. GmKRP2a was overexpressed in the soybean hairy root *via Agrobacterium rhizogenes*-mediated transient transgenic system to elucidate the role of GmKRP2a during root development. After initially selecting the positive transgenic roots with hygromycin resistance, the same length transgenic roots of the control and overexpressing lines were transferred to the new medium to investigate the growth rate. The presence of the construct and its expression level were checked using PCR and qPCR, respectively (**Supplementary Figure 4**). As shown in **Figures 4A, B**, the transgenic soybean hairy roots exhibited a 35–50% length increase relative to the controls after 7 days. To further investigate gene function, the same construct was used to transformed into *Arabidopsis* to generate stable transgenic lines. As shown in **Figures 4C, E**, three independent transgenic *Arabidopsis* lines with single insert copies were used for further phenotype analysis. An average root growth promotion of 29–34% and 24–37% hypocotyl promotion under dark conditions were observed in *GmKRP2a* overexpression lines (**Figures 4D, F**). These results indicate that GmKRP2a is a positive regulator of soybean root elongation.

**Figure 5:**
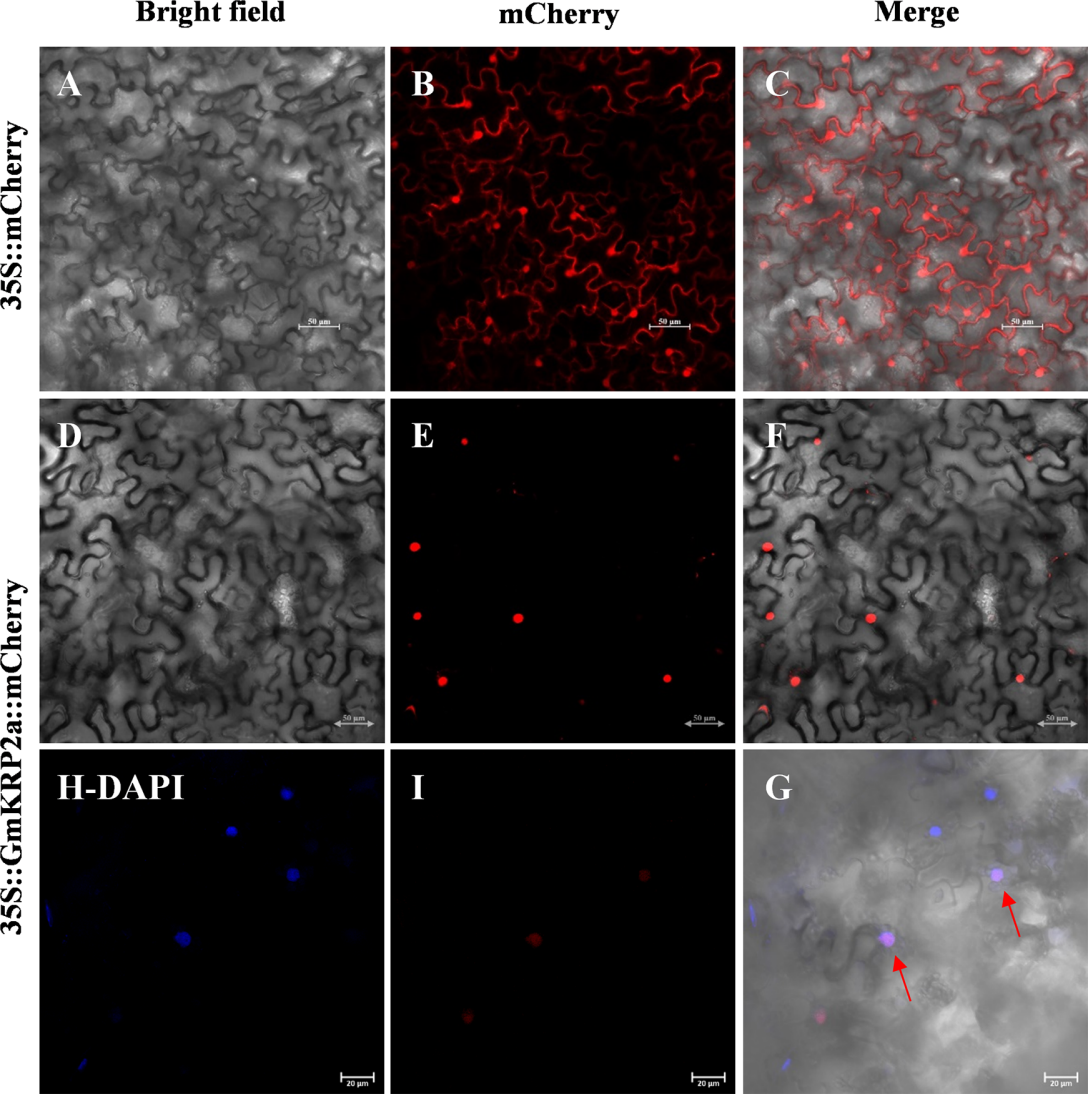
Subcellular localization of the GmKRP2a protein in tobacco leaf hypodermal cells. **(A-C)** Confocal scanning microscopic images show localization of mCherry proteins to cell membrane and nucleus. **(D-F)** Confocal scanning microscopic images show localization of GmKRP2a-mCherry fusion proteins to nucleus. **(H-G)** Epidermal cells of tobacco expressing GmKRP2a-mCherry under DAPI blue florescence. Left panel: bright field image, middle panel: mCherry channel, right panel: merged. Bars = 50 μm.

In addition, the subcellular localization of GmKRP2a was investigated using the transient expression of 35S::GmKRP2a–mCherry in tobacco leaves. The results showed clear nuclear fluorescence in the tobacco cells (**Figures 5D, F**) compared with empty vector control (**Figures 5A–C**). The nuclei were stained with DAPI, which allows detection of nuclear DNA. Colocalization of mCherry (red) with the DAPI (blue) was shown by red arrows (**Figures 5G, H**). Furthermore, the subcellular localization of GmKRP2a was further validated using transgenic *Arabidopsis* carrying 35S::GmKRP2a–GFP. The overlap between DAPI and GFP fluorescence confirmed that GmKRP2a was a nuclear protein, which is consistent with the localization prediction and the function in cell cycle (**Supplementary Figure 5**).

### Gain of GmKRP2a function increases DNA content and promotes cell expansion

**Figure 6:**
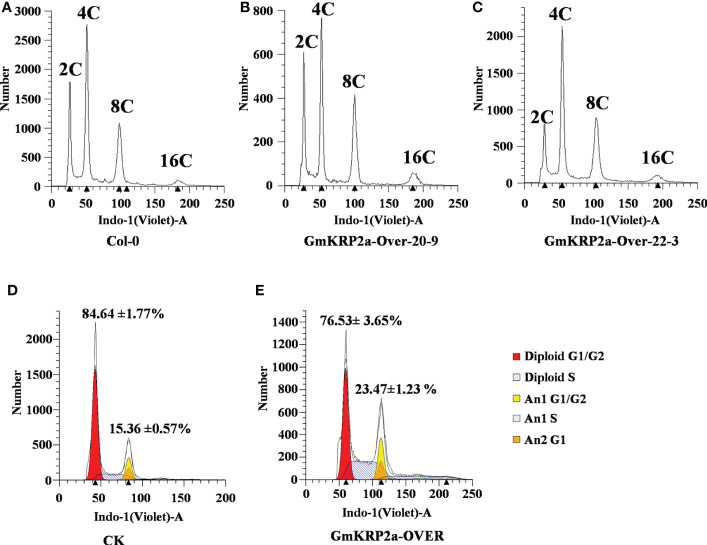
Overexpression of GmKRP2a leads to an increased progression through endoreduplication. **(A-C)** DNA content distribution of nuclei isolated from the first cauline leaf of wild type (Col-0) and two independent GmKRP2a-overexpressing Arabidopsis transgenic lines. **(D-E)** Representative ploidy histograms for nuclei isolated from empty vector control and GmKRP2a-over lines in soybean hairy root. 2 cm hairy root from tip were used for analysis.

To address the gene function of GmKRP2a in the cell cycle, the ploidy level in rosette leaves of GmKRP2a-Over and Col-0 plants was determined using flow cytometric analysis. The proportion of cells with an increased DNA content in GmKRP2a-Over transgenic plants was higher than that of Col-0 (**Figures 6A–C**). Moreover, the maximal nuclear DNA level reached 16C in the GmKRP2a-overexpressing roots, whereas the cells of the WT roots displayed an essentially 2C and 4C DNA content distribution. The endoreduplication level was increased in these GmKRP2a-overexpressing plants. Furthermore, the distribution of 2C nuclei was shifted proportionally to the corresponding 4C, 8C, and 16C nuclei. GmKRP2a-overexpressing hairy roots showed an overall higher endoreduplication than the control roots (**Figures 6D, E**, P < 0.01), which strongly suggests that GmKRP2a inhibits the cell cycle during the G2/M phase.

**Figure 7:**
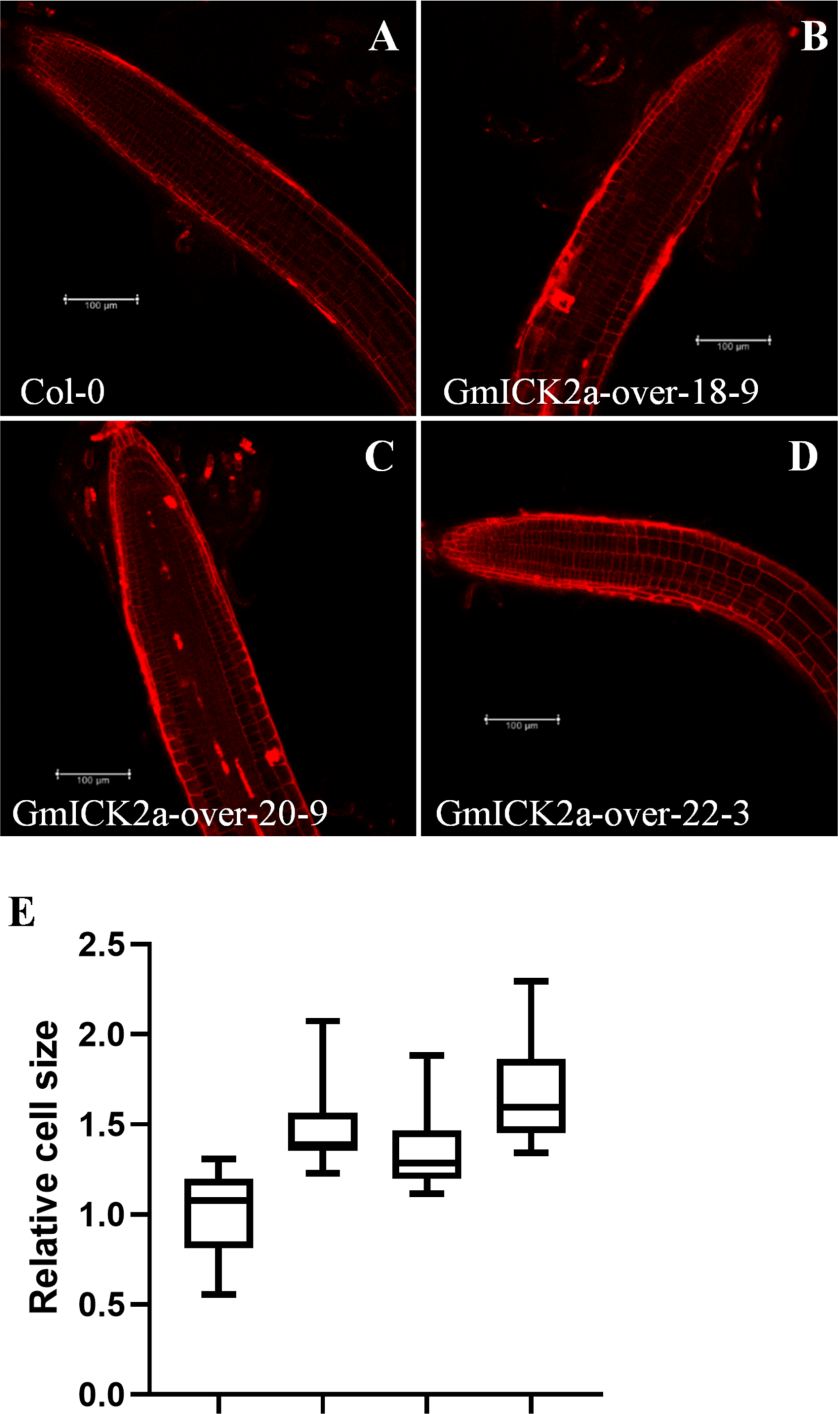
Confocal microscopic images of root and cell numbers in WT and three 35S:GmKRP2a-GFP transgenic independent lines which stained with propidium iodide. **(A)** 10-day old post-germination root tip, above the meristematic tip, and elongation zone in WT root. **(B-D)** 10-day old post-germination root tip, above the meristematic tip, and elongation zone in three independent GmKRP2a-over Arabidopsis transgenic root. **(E)** Comparison of cell size between WT and three 35S:GmKRP2a-GFP transgenic independent lines (relative ratio = value of mutant/value of WT). Six cells were measured for each root tip and over 30 seedlings of each transgenic lines were used. Columns marked with * indicate significant differences (Student’s *t*-test, P < 0.05).

Since we previously found that the root elongation was promoted, whereas the cell division was inhibited in GmKRP2a-overexpresion plants, the question on whether GmKRP2a influences cell expansion were further addressed. Therefore, the roots from the GmKRP2a-overexpressing transgenic lines and the WT were observed using a fluorescence microscope after propidium iodide staining. The cell size of the cortex cells in the proximal meristem was more promoted than that of Col-0 (**Figures 7A–D**). The cell size was determined using ImageJ software. The overall increase in size was observed in the GmKRP2a-overexpressing *Arabidopsis* lines, which induced 30–50% cell expansion (**Figure 7E**).

### *GmKRP2a* overexpression may decrease the number or activity of ribosomes

High-quality RNA-seq was performed to investigate the transcriptomes of 35S::GmKRP2a hairy root lines and the empty vector control for the genome-wide discovery of differentially expressed genes (DEGs) (**Supplementary Table 5**). A total of 1291 DEGs were identified with the criteria |log2FC|≥1 and p<0.05 (**Supplementary Table 6**). Among these, 989 and 302 DEGs were up- and downregulated, respectively. A subset of 18 DEGs was selected for the validation of the expressions using qPCR, and the results showed that 18 DEGs displayed expression patterns consistent with the expression levels obtained from RNA-seq analysis (**Supplementary Figure 6**). Interestingly, there were 102 genes encoding eukaryotic small subunit ribosomal RNA among the downregulated DEGs. Moreover, no SSU_rRNA were upregulated in 35S::GmKRP2a hairy root. In addition, three 5.8S ribosomal RNA were significantly downregulated. These results may indicate that GmKRP2a overexpression decreases the number or activity of ribosomes by downregulating the gene expression of ribosomal small subunit RNA, which further results in a decrease in the level of protein translation in cells and a stagnation of the cell cycle in the G2/M phase.

Furthermore, all functionally annotated transcript sequences were selected and assigned to the reference canonical pathways in KEGG. A total of 127 genes were distributed to 14 different pathways in the KEGG database (p<0.05). KEGG annotation analysis revealed that the plant–pathogen interaction pathway was the most enriched KEGG term with the highest level of gene representation (48 transcripts, **Supplementary Table 7**), followed by lipid metabolism, energy metabolism, and signal transduction (**Supplementary Figure 7**). This result suggests that increased endoreduplication and inhibited cell division may correlate with pathogen resistance in plants.

## Discussion

### Characterization of the soybean *KRP* gene family

As a crucial negative regulator of the cell cycle, the *KRP* genes have been identified, and a considerable amount of research have been studied in several plant species and have been demonstrated to play significant roles on plant development and in response to various stressors (Polyn et al., 2015; Kumar and Larkin, 2017; Vieira and de Almeida Engler, 2017; Xiao et al., 2017; Sahu et al., 2021). In this study, a comprehensive analysis of genes encoding KRP proteins in the soybean genome was performed, resulting in the identification of nine KRP family members. The phylogenetic analysis and evolutionary process of the KRP family laid the foundation for further functional investigation of *KRP* genes. Among the 10 species analyzed in this paper, soybean contains the largest number of *KRP* genes. The soybean genome underwent two rounds of whole-genome duplication, which occurred 59 and 13 million years ago (Schmutz et al., 2010). The expansion of *GmKRP* genes has arisen more recently due to soybean-specific duplication. This result may indicate that GmKRPs play important roles in regulating biological processes, which need to be demonstrated further. As expected, GmKRPs were grouped more closely to legume KRPs compared to *Arabidopsis* and monocotyledonous plants.

The gene structures were highly conservative in all 10 species. Plant KRPs shared a low sequence identity with non-plant KRPs. The CID domain at the C terminal, which is required for interacting with the CDKA protein and inhibiting its activity in plants, was the most conserved motif among all plant KRP proteins, (Wang et al., 1998; Zhou et al., 2003b; Torres Acosta et al., 2011). Sequence alignment of the KRP family in this study confirmed the presence of the conserved C-terminal domain in soybean, whereas the randomness of the N-terminal sequence was much higher than that of the C-terminal. The N-terminal region increases KRP1 instability, and the central domain is responsible for nuclear localization (Schnittger et al., 2003; Zhou et al., 2003a). It was considered that the diversity of the N-terminal amino acids caused a diversity of different KRP functions.

The identification of SNPs and their potential effects or functional annotations in one gene family is crucial for studying the genetic basis of phenotypic differences (Schacherer et al., 2009). Several gene-based SNPs have been reported for their roles in controlling traits, such as grain filling, plant height, amylose content, and grain structure in rice and maize (Tanaka et al., 1995; Monna et al., 2002; Wang et al., 2008; Godínez-Palma et al., 2017). In this study, only one non-synonymous substitution of GmKRP2a was identified in one *Glycine soja* line (PI 562534) across 775 soybean germplasm resources, which resulted in amino acid conversion from Ala^151^ to Thr^151^. This amino acid conversion may affect the gene function because it is in the conserved cyclin-binding domain. Sequence variants that affect gene functions can be used to design functional molecular markers (Andersen and Lubberstedt, 2003). Therefore, this identified SNP can be further used to develop functional markers.

### Regulation of the expression level and roles of KRP in root development

The initiation and division of root or leaf primordium or the formation and activation of the meristem during plant tissue development is largely determined by the transition between cell division and cell expansion during the cell cycle (Inze and De Veylder, 2006; Inagaki and Umeda, 2011). These processes and transitions are regulated by a protein complex composed of highly conserved regulators of CYC, CDK, and CKI. However, plant growth involves the integration of many environmental and endogenous signals in which hormones act as an internal signal involved in the determination of plant size. GA, auxin, and brassinosteroids affect proportional changes in organ growth rates (Himanen et al., 2002; Verkest et al., 2005b; Achard et al., 2009; González-Garcia et al., 2011; Zhao et al., 2017; Li et al., 2022). In this study, the promoter region of *GmKRP2a* contained many methyl jasmonate, auxin, gibberellin, and abscisic acid response elements. Overexpression of *GmKRP2a* appeared to restrain cell division and enhanced the levels of endoreduplication. Therefore, it could be hypothesized that one of the pathways that hormone control the cell cycle by modulating the expression levels of *GmKRP2a*.

Drought is one of the main unfavorable factors affecting the growth and development, seed yield, and quality of soybean. The annual loss of soybean yield and quality caused by drought is immeasurable. The trait of deeper roots allows the plant to search for additional soil water while maintaining higher water-use efficiency under drought stress conditions (Calleja-Cabrera et al., 2020). Here, overexpression of *GmKRP2a* in both soybean transgenic hairy root and stably transformed *Arabidopsis* plants leads to a significant increase in root elongation, which provides an experimental basis for how to use *GmKRP2a* to promote the development of roots and improve the drought resistance/tolerance of soybean. Understanding the role of cell cycle regulators during plant development will not only clarify the molecular mechanism, but also guide the agricultural practice. In the future, how GmKRP2a connects root development with environmental signals to feed into cell cycle regulation and better targeted in specific cells should be addressed.

However, the role of KRP is very complicated, and its function in different tissues may be different. For example, overexpression of *NtKIS1a* in *Arabidopsis* induces modified plant morphology with smaller organs that contain larger cells, similar to *Arabidopsis* plants overexpressing KRP genes (Wang et al., 1998; De Veylder et al., 2001; Zhou et al., 2002). *NtKIS1a* and *NtKIS2* showed obvious differences in expression patterns during both the cell cycle and plant development (Jasinski et al., 2002; Jasinski et al., 2003). Overexpressed Orysa; *KRP1* reduced cell production during leaf development and dramatically reduced seed filling (Barroco et al., 2006). Overexpression of *OsiCKI6* reduces the seed-setting rate, partially due to the poorly developed pollen, and results in a reduced cell number, but larger cells in the leaf and stem coincide with the transgenic plant dwarfed phenotype (Yang et al., 2011). In the current study, no obvious phenotype was observed in GmKRP2a-overexpressing *Arabidopsis thaliana* aboveground tissue (data not shown).

### GmKRP2a may play an important role in pathogen resistance

It has been reported that KRP3, KRP5, KRP6, and KRP7 play roles in the root knot nematodes resistance by inhibiting the size of giant cells or affecting nematode development and offspring (Vieira et al., 2012; Vieira et al., 2014; Coelho et al., 2017). Hamdoun et al. (2016) reported that SIM and SMR1 act in innate immunity in *Arabidopsis*. These results suggest that precise cell cycle control is not only critical for plant development but also important for plant–pathogen response. In this study, overexpressed *GmKRP2a* induced the expression level of many plant–pathogen-related genes. Among them, *BAK1* (Glyma.05G119500) and *FLS2* (Glyma.05G128200) were both induced in the GmKRP2a overexpressing lines. The BAK1 and FLS2 complex acts as a receptor for several LRR-type PRRs (Chinchilla et al., 2007; Heese et al., 2007; Wang et al., 2020). Therefore, we propose that GmKRP2a may play a role in the plant defense response. Further investigation and characterization of the GmKRP2a function will provide a better understanding of crosstalk between cell cycle regulation and plant defense.

## Conclusion

In the present study, we identified nine *GmKRP* genes in the soybean genome. We analyzed their phylogeny, gene structure, tissue expression pattern, SNPs, and conserved motifs to determine their evolutionary relationships with other plant species. Furthermore, *GmKRP2a*, one of the KRP genes, was cloned for gene function analysis. Based on the findings presented in the current study, it is reasonable to conclude that the soybean cyclin-dependent kinase inhibitor gene *GmKRP2a* plays an important role in the regulation of root development by inhibiting the cell cycle, which leads to enhanced endoreduplication levels and promotes cell expansion. These findings will serve as a strong foundation for future research on root elongation with cell cycle modification. Notably, the GmKRP gene family should be connected to genes encoding cyclin-dependent protein kinases, and cyclin proteins that are involved in endocycles in soybean. In the future, the interaction network of GmKRP2a should be explored to further elucidate its function.

## Data availability statement

The original contributions presented in the study are publicly available. This data can be found here: NCBI, PRJNA907965.

## Author contributions

LS: conceptualization and writing-original draft. BG and LC: methodology and analysis. BG and LC: resources and validation. LC, LD, CY, XG, and JZ: formal analysis and investigation. LS: funding and supervision. LS, LZ, and BG: writing, review, and editing. All authors contributed to the article and approved the submitted version.

## Supplementary material

Supplementary Figure 1 Phylogenetic relationships, conserved motif composition and schematic diagram for intron/exon gene structure of the KRP proteins from ten different plant species. Left panel: An unrooted phylogenetic tree constructed with the neighbor-joining method. Mid panel: Distribution of conserved motifs in KRP proteins using the MEME web server. The differently colored boxes represent different motifs and their positions in each KRP protein sequence. Right panel: gene structure of KRP proteins. The black line, green boxes and yellow boxes indicate introns, UTRs, and exons, respectively. Gene models are drawn to scale as indicated on bottom.

Supplementary Figure 2 Amino acid alignment of nine GmKRP family members and conserved motif sequences. **(A)** Amino acid alignment of GmKRPs. The numbers on the right indicate amino acid position. Shared conserved amino acid residues are in yellow background. Gaps, indicated by dashed lines are introduced for optimal alignment. Alignments were performed using ClustalW. PEST domains are marked by the blue underline. **(B)** Sequence logos of ten motifs identified by MEME.

Supplementary Figure 3 GO enrichment analysis of the GmKRP co-expression genes in *Glycine max*. **(A)** GO enrichment analysis in biological process, **(B)** GO enrichment analysis in molecular function terms.

Supplementary Figure 4 qPCR analysis of GmKRP2a relative expression level. (**A**) qPCR analysis of three GmKRP2a overexpression transgenic soybean hairy root samples confirming ~400-fold higher expression level than that of control vector. Error bars represent standard deviation (SD) of three replicates. * P<0.05 for OX lines relative to the WT or empty vector control. (**B**) qPCR analysis of three GmKRP2a overexpression transgenic *Arabidopsis* lines confirming ~10^6^-fold higher expression level than that of wild-type (Col-0).

Supplementary Figure 5 Subcellular localization of GmKRP2a-GFP protein in transgenic Arabidopsis root. **(A)** Bright field, **(B)** DAPI staining exhibiting intact nuclei, **(C)** Visualization of GmKRP2a-GFP expression, **(D)** Merged image with bright field. Bars = 50 μm.

Supplementary Figure 6 Validation of the RNA-Seq data by qPCR analysis. Linear regression plots for eighteen randomly piked genes. Pearson’s correlation coefficients (r) were calculated.
