# Supplementary figures and images for: Characterization of the soybean KRP gene family reveals a key role for GmKRP2a in root development
Source: Front Plant Sci. 2023 Jan 27;14:1096467. doi: 10.3389/fpls.2023.1096467 (PMC9911667; doi:10.3389/fpls.2023.1096467)

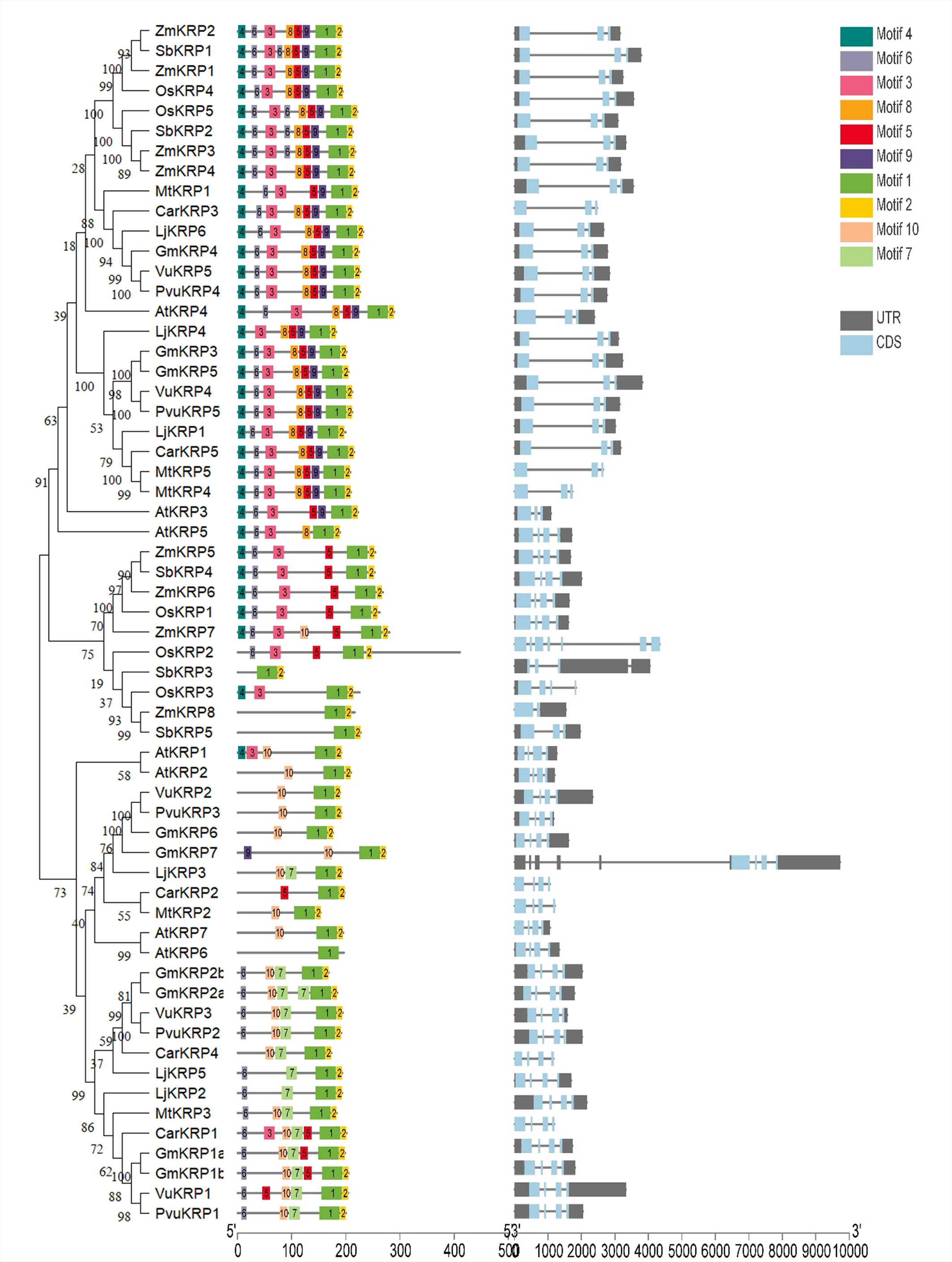

Supplement: Supplementary Figure 1 — Phylogenetic relationships, conserved motif composition and schematic diagram for intron/exon gene structure of the KRP proteins from ten different plant species. Left panel: An unrooted phylogenetic tree constructed with the neighbor-joining method. Mid panel: Distribution of conserved motifs in KRP proteins using the MEME web server. The differently colored boxes represent different motifs and their positions in each KRP protein sequence. Right panel: gene structure of KRP proteins. The black line, green boxes and yellow boxes indicate introns, UTRs, and exons, respectively. Gene models are drawn to scale as indicated on bottom. [file Image_1.tif]

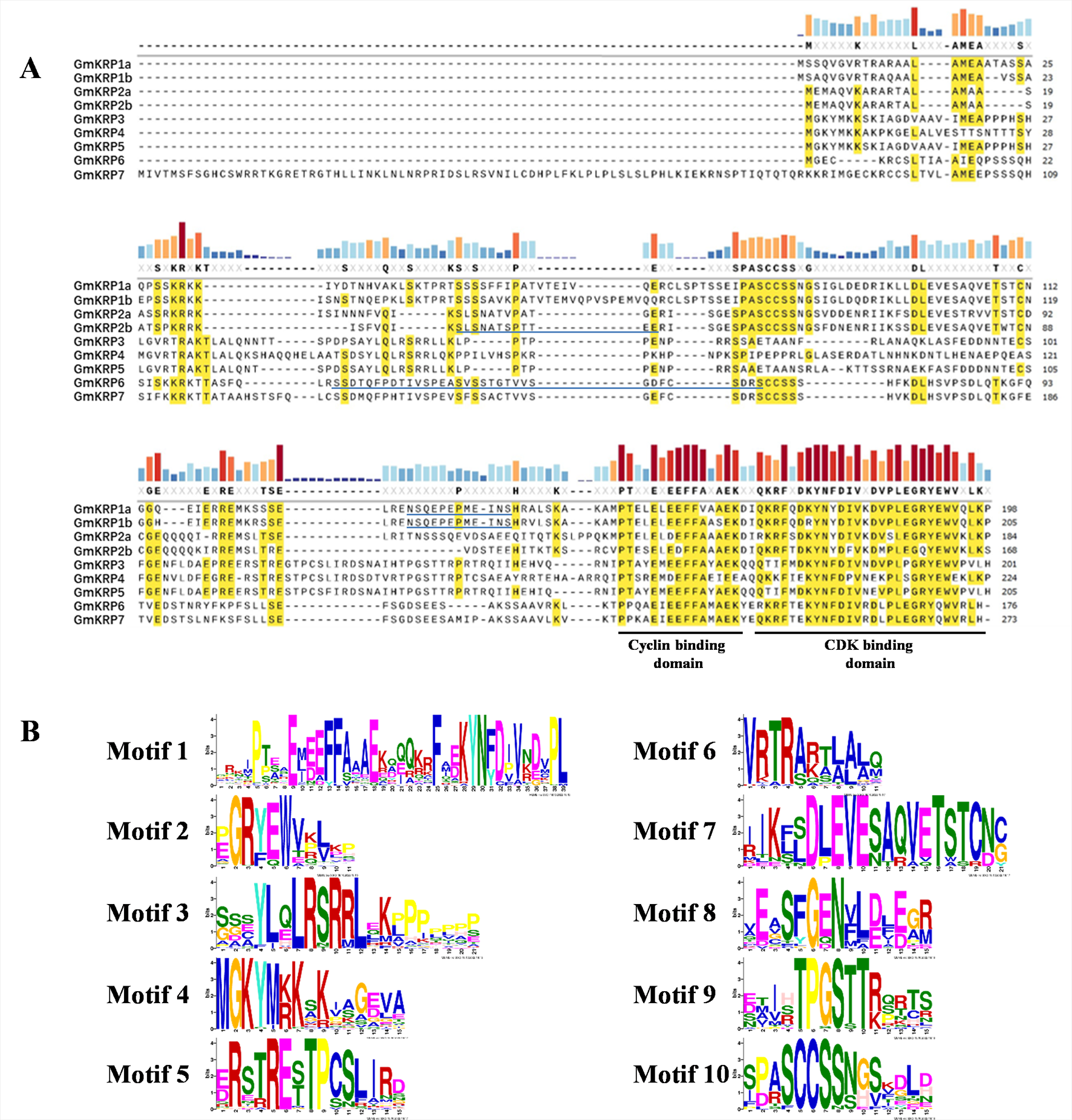

Supplement: Supplementary Figure 2 — Amino acid alignment of nine GmKRP family members and conserved motif sequences. (A) Amino acid alignment of GmKRPs. The numbers on the right indicate amino acid position. Shared conserved amino acid residues are in yellow background. Gaps, indicated by dashed lines are introduced for optimal alignment. Alignments were performed using ClustalW. PEST domains are marked by the blue underline. (B) Sequence logos of ten motifs identified by MEME. [file Image_2.tif]

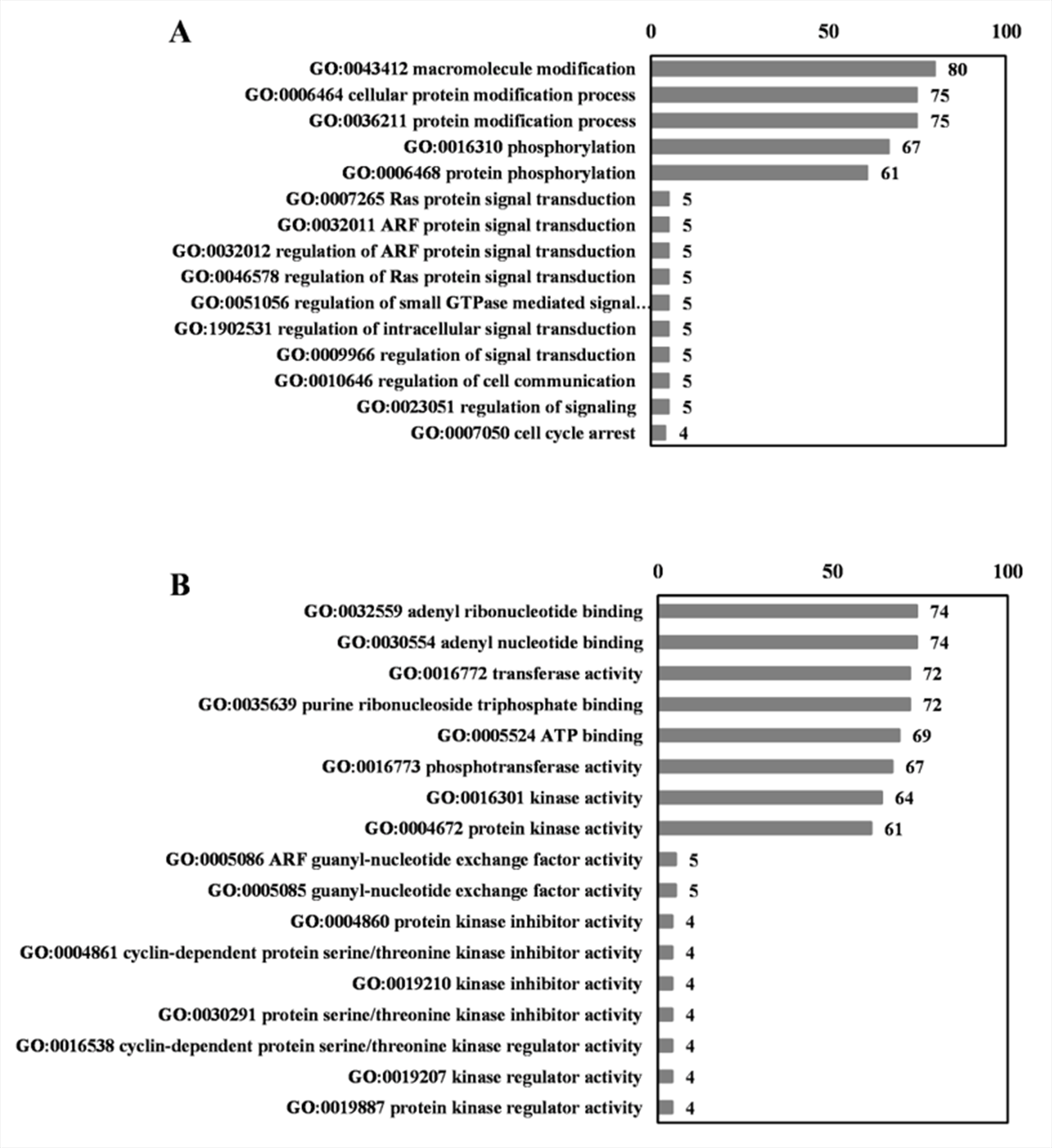

Supplement: Supplementary Figure 3 — GO enrichment analysis of the GmKRP co-expression genes in Glycine max. (A) GO enrichment analysis in biological process, (B) GO enrichment analysis in molecular function terms. [file Image_3.tif]

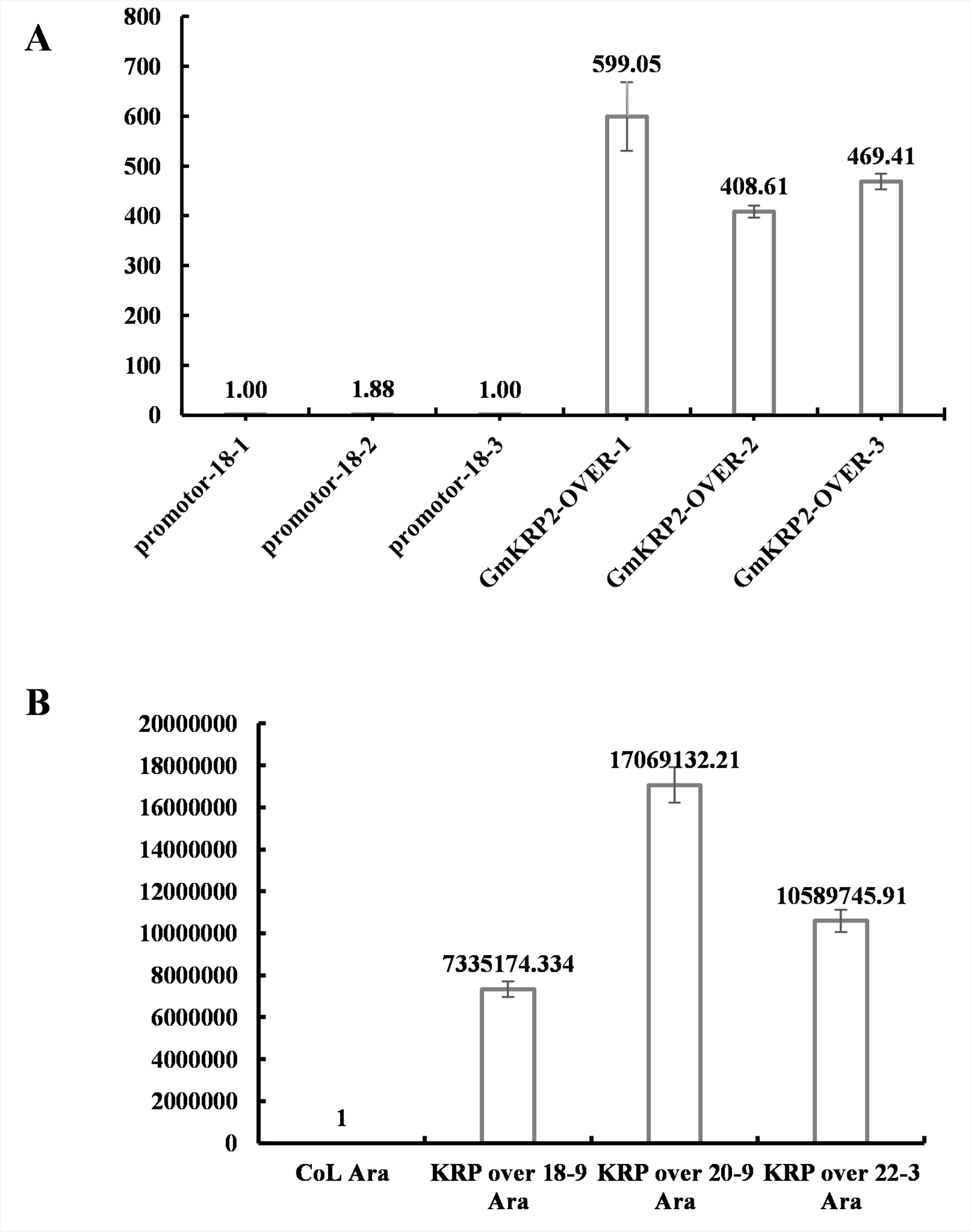

Supplement: Supplementary Figure 4 — qPCR analysis of GmKRP2a relative expression level. (A) qPCR analysis of three GmKRP2a overexpression transgenic soybean hairy root samples confirming ~400-fold higher expression level than that of control vector. Error bars represent standard deviation (SD) of three replicates. * P<0.05 for OX lines relative to the WT or empty vector control. (B) qPCR analysis of three GmKRP2a overexpression transgenic Arabidopsis lines confirming ~106-fold higher expression level than that of wild-type (Col-0). [file Image_4.tif]

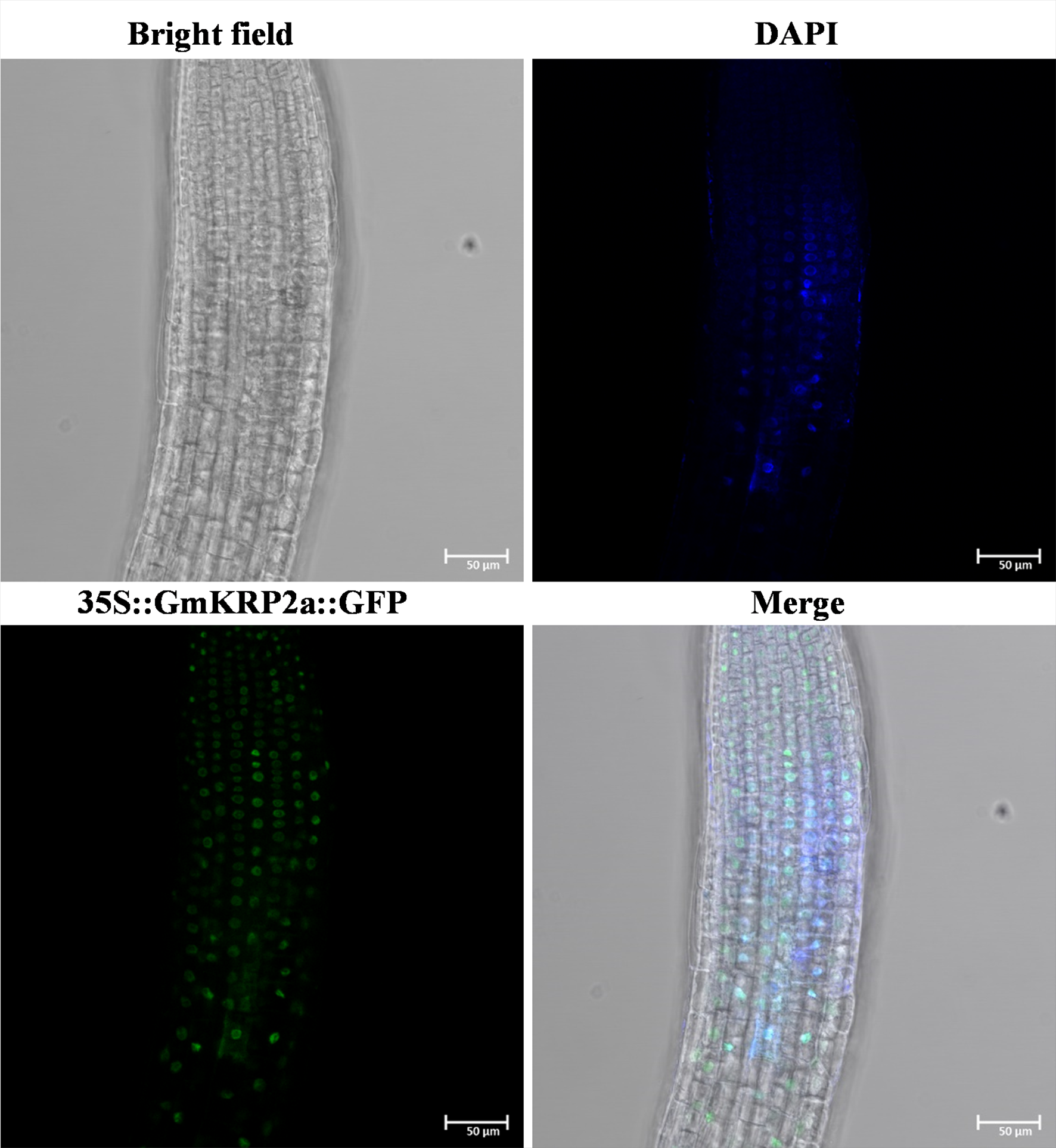

Supplement: Supplementary Figure 5 — Subcellular localization of GmKRP2a-GFP protein in transgenic Arabidopsis root. (A) Bright field, (B) DAPI staining exhibiting intact nuclei, (C) Visualization of GmKRP2a-GFP expression, (D) Merged image with bright field. Bars = 50 μm. [file Image_5.tif]

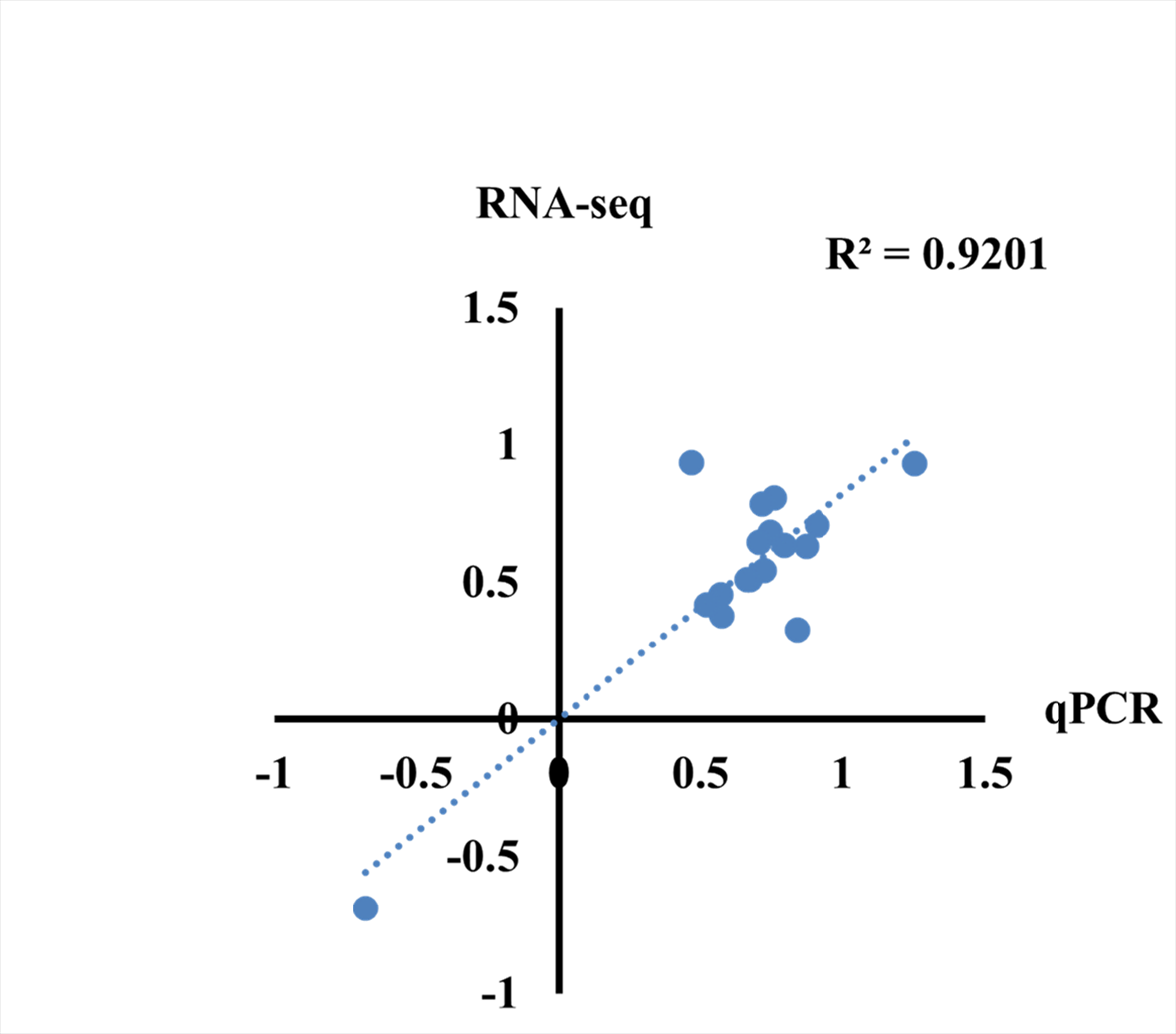

Supplement: Supplementary Figure 6 — Validation of the RNA-Seq data by qPCR analysis. Linear regression plots for eighteen randomly piked genes. Pearson’s correlation coefficients (r) were calculated. [file Image_6.tif]

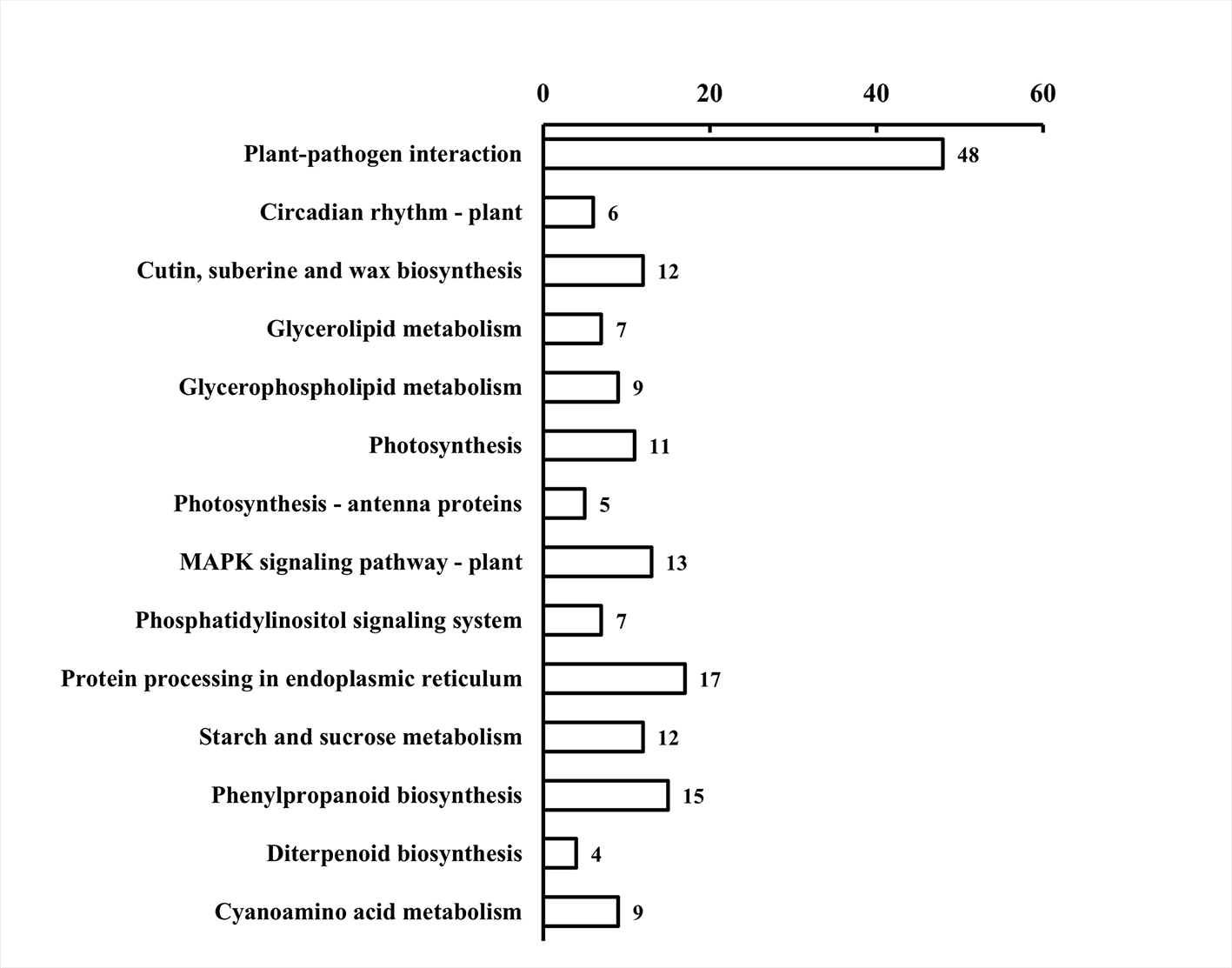

Supplement: Supplementary file 7 [file Image_7.tif]
